# Recurrent Hormone Receptor–Positive Breast Cancer With Multiple Sites of Distant Metastasis: A Case Report and Review of the Literature

**DOI:** 10.1155/ijbc/5325167

**Published:** 2025-09-29

**Authors:** Lu Li, Hui Liu, Xiangxin Huang, Haojun Luo

**Affiliations:** ^1^Department of Thyroid and Breast Surgery, The Second Affiliated Hospital of Chongqing Medical University, Chongqing, China; ^2^University of the Chinese Academy of Sciences Chongqing Renji Hospital, Chongqing, China

**Keywords:** bone metastasis, pseudoprogression, recurrent breast cancer

## Abstract

Breast cancer is one of the most common malignancies and the leading cause of cancer-related mortality among women worldwide, with hormone receptor–positive (HR+) breast cancer being the most common subtype. Current guidelines recommend endocrine therapy as the first-line treatment for HR+, human epidermal growth factor Receptor 2–negative breast cancer. In this case report, we describe a patient with HR+ breast cancer who developed bone and liver metastases after breast cancer surgery. We document the disease progression from initial treatment to managing local recurrence and treating distant metastasis using salvage chemotherapy combined with endocrine therapy. Importantly, following denosumab treatment, the patient experienced a bone flare; this presented as increased radionuclide uptake on bone scans, but was later confirmed as pseudoprogression. Furthermore, we note changes in the patient's pathology as the disease progresses.

## 1. Background

Hormone receptor–positive (HR+) breast cancer accounts for approximately 60%–80% of all breast cancer cases [1]. Endocrine therapy is the recommended first-line therapy for HR+, human epidermal growth factor Receptor 2–negative (HER2−) breast cancer [2]. Endocrine therapy suppresses tumor cell growth by reducing the level of estrogen or inhibiting its activity, offering advantages such as minimal adverse events, excellent tolerance and treatment compliance, and good efficacy. Common endocrine therapy medications include tamoxifen (TAM), aromatase inhibitors (AIs), ovarian function suppression (OFS), cyclin-dependent Kinase 4/6 (CDK4/6) inhibitors, selective estrogen receptor (ER) degraders, and selective ER modulators. Chemotherapy and poly ADP-ribose polymerase inhibitors are also alternative treatment options. Compared to hormone receptor–negative breast cancer, HR+ breast cancer is associated with longer overall survival [3]. As the disease progresses, pathological characteristics change, necessitating adjustments in treatment regimens. The bone is the most common site for breast cancer metastases, occurring in up to 68.9% of metastatic breast cancer cases, followed by the lung (32.1%) and the liver (27.7%) [3]. This article reports a case of HR+ breast cancer recurrence in the chest wall 11 years postmastectomy, with multiple metastases in the bone and liver, and reviews the literature.

## 2. Case Report

**Figure 1:**
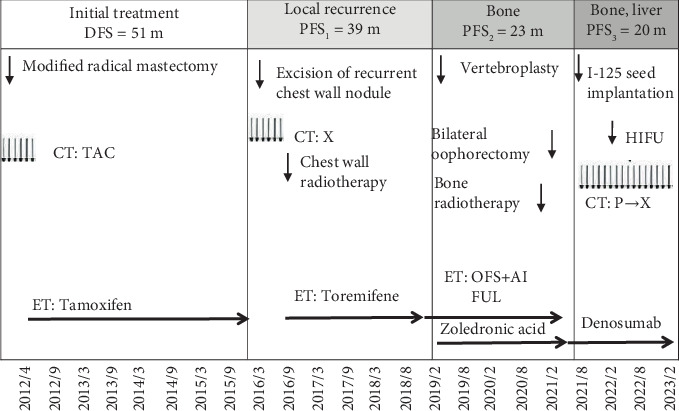
Overview of the patient's clinical course. ER, estrogen receptor; HER2, human epidermal growth factor Receptor 2; PR, progesterone receptor; DFS, disease-free survival; PFS, progression-free survival; CT, chemotherapy; TAC (T, docetaxel; A, pirarubicin; C, cyclophosphamide); ET, endocrine therapy; OFS, ovarian function suppression; AI, aromatase inhibitor; FUL, fulvestrant; X, capecitabine; P, albumin-bound paclitaxel; HIFU, high-intensity focused ultrasound.

In March 2012, a 38-year-old premenopausal patient with invasive ductal carcinoma of the left breast underwent a modified radical mastectomy. She had no family history of breast cancer. Pathology report showed ER (++) 50%, progesterone receptor (PR) (+++) 75%, HER2−, and Ki-67 20% (+) (Table 1). This patient was diagnosed with luminal B HER2− breast cancer (pT2N1M0, Stage IIB) and received adjuvant chemotherapy with TAC for six cycles (120 mg docetaxel + 60 mg epirubicin + 800 mg cyclophosphamide ivgtt q3w) and adjuvant endocrine therapy with TAM (10 mg PO bid) (Figure 1).

In July 2016, at the age of 42, the patient experienced a recurrence in the left chest wall and underwent wide excision of the chest wall lesion. Pathological report showed ER (+) 10%, PR (−), HER2 (−), and Ki-67 (+) 15% (Table 1). Following surgery, the patient received capecitabine (1.5 mg PO bid, d1–14 q3w) for six cycles, followed by chest wall radiotherapy (CWRT) at a dose of 2 Gy × 25, 50 Gy in total. The oral endocrine treatment was switched to letrozole (60 mg PO qd) after surgery. The DFS (disease-free survival) was 51 months (Figure 1).

In October 2019, the patient reported pain in the left lower limb. CT and MRI revealed a lesion in the T8 vertebral body. Percutaneous vertebroplasty and biopsy were performed. The pathological report showed ER (−), PR (+) 5%, HER2 (−), and Ki-67 (+) < 1% (Table 1), confirming bone metastasis from breast cancer. The patient was treated with leuprorelin (3.75 mg SC q28d) and letrozole (2.5 mg PO qd), as well as zoledronic acid (4 mg ivgtt q28d) for bone metastasis. The progression-free survival (PFS) was 39 months (Figure 1).

In January 2020, the patient reported left hip pain, and ECT revealed a patchy radioactive concentration in the left ilium. Treatment remained unchanged at the time. In March 2021, the patient's hip pain worsened, and MRI showed an increased extent of the lesion in the left hip bone, with possible metastases in the L2, L3, L5, S1, and S4 vertebrae. Laparoscopic bilateral oophorectomy was performed, followed by fulvestrant administration (250 mg IM q4w). In July 2021, the patient underwent intensity-modulated radiation therapy at 3 Gy × 10 (totaling 30 Gy) to the vertebrae and 3 Gy × 15 (totaling 45 Gy) to the hip joint. The patient's treatment regimen was further modified, switching from zoledronic acid to denosumab (120 mg SC q4w) (Figure 1).

In September 2021, ECT indicated an increased range of metastases, including new consolidations in the right scapula, multiple areas in the spine, and the femur and left ilium. Additionally, a liver mass was detected on the CT scan. Liver biopsy confirmed infiltrating poorly differentiated adenocarcinoma, ER (−), PR (−), HER2 (1+), and Ki-67 (+) 40% (Table 1), indicating liver metastasis from breast cancer. The patient received radioactive particle implantation (iodine-125) for liver metastases and was also treated with albumin-bound paclitaxel (260 mg/m^2^ ivgtt q3w) for maintenance therapy over a year. The disease progressed, with a PFS of 23 months (Figure 1).

In March 2022, abdominal MRI with dynamic enhanced diffusion-weighted imaging showed a significant reduction in the liver lesion compared to previous scans. The patient underwent palliative high-intensity focused ultrasound therapy for metastatic liver cancer.

In October 2022, ECT revealed no new bone metastases, and the existing lesions remained unchanged. The treatment was switched to capecitabine (1000 mg bid) for metronomic chemotherapy while continuing treatment with denosumab.

Last follow-up: May 2023. No signs of tumor progression or new metastases were found.

## 3. Discussion

### 3.1. Changes in Treatment Strategies

The patient initially presented as a premenopausal woman undergoing a modified radical mastectomy (left). Pathological confirmation revealed luminal B HER2− breast cancer (pT2N1M0, Stage IIB), and chemotherapy was initiated. Anthracycline-based chemotherapy is one of the preferred regimens for HR+ HER2− early breast cancer. A meta-analysis based on 123 randomized trials showed a 33% reduction in 10-year cancer mortality with anthracycline-based, taxane-based, and alkylating agent–based chemotherapy compared to no chemotherapy [2]. In this case, the patient received TAC chemotherapy, combining anthracyclines and taxanes, which was guideline recommended at the time. The patient had 3/18 axillary lymph node metastases, was younger than 40 years old, and had indications for postoperative radiotherapy, but did not receive it due to personal reasons. Adjuvant radiation therapy for early stage breast cancer commonly targets the chest wall and supraclavicular lymph nodes. Meta-analyses have demonstrated that postoperative radiation therapy significantly improves local control rates and DFS [4, 5].

When endocrine therapy was initiated with TAM, the patient was premenopausal, and the benefit of OFS in premenopausal patients was uncertain. According to data from the Suppression of Ovarian Function Trial (SOFT) and Tamoxifen and Exemestane Trial, compared with TAM, the combination regimen of OFS and AI can significantly reduce distant recurrence and death for patients at clinical high risk [6]. Moreover, for early stage high-risk HR+ HER2− breast cancer, CDK4/6 inhibitors have been shown to reduce recurrence and metastasis. The monarchE trial results showed that adding abemaciclib, a selective CDK4/6 inhibitor, to endocrine therapy provided a sustained benefit compared to endocrine therapy alone, with a 5-year absolute improvement in invasive DFS rates of 7.6% (hazard ratio: 0.680, 95% CI: 0.599–0.772, median follow-up: 54.0 months) [7].

Following local recurrence, the patient underwent resection of the recurrent lesion and received local radiation therapy. Subsequent studies from the Danish Breast Cancer Cooperative Group involving 535 cases of local recurrence after mastectomy with chemotherapy and radiation therapy showed a complete response rate of only 46% and a local control rate of only 11% after 2 years with systemic therapy [8]. In contrast, the combination of surgery and radiation therapy achieved a complete response rate of 96% and a local control rate of 45% after 2 years, indicating a higher local control rate with local treatment compared to systemic treatment for local recurrent lesions. A retrospective multicenter study by Zhao et al. evaluated 205 patients with isolated chest wall recurrence (ICWR) after mastectomy and compared outcomes between different local treatment strategies. Among these, patients who did not receive radiotherapy had a 5-year locoregional recurrence-free survival (LRRFS) rate of only 18.2%, while those treated with CWRT alone achieved a significantly higher 5-year LRRFS rate of 47.1% (*p* < 0.001) [9]. These results indicate that CWRT substantially improves locoregional control compared to no radiotherapy in patients with ICWR after mastectomy.

In July 2016, the patient was 42 years old, still premenopausal, and remained sensitive to endocrine therapy. The recurrent lesion was still ER-positive. The preferred endocrine treatment for recurrent or metastatic breast cancer in premenopausal patients is ovarian suppression or ablation combined with endocrine therapy: ovarian suppression or ablation plus TAM (for those who have never used TAM or discontinued it more than 12 months ago) or ovarian suppression or ablation plus an AI (for those who have previously used TAM). However, since the patient refused ovarian resection and could not afford ovarian function suppressors, selective ER modulators were considered viable alternatives. Therefore, endocrine therapy with toremifene was initiated instead. Considering the inadequate intensity of endocrine therapy, capecitabine was administered as rescue chemotherapy before initiating endocrine therapy. With an ER-positive rate of only 10% and negative PR, there was also a rationale for adding chemotherapy to the treatment regimen.

Advanced HR+/HER2− breast cancer includes breast cancer that cannot be surgically removed or has spread from the axilla to other organs [1]. In recent years, advances in treatment strategies have led to improved patient outcomes [10]. For HR+/HER2− metastatic breast cancer, endocrine therapy combined with CDK4/6 inhibitors is the current preferred treatment, and AIs have demonstrated superiority over TAM [11, 12]. Fulvestrant, a selective ER degrader, has demonstrated efficacy as a first-line treatment for postmenopausal HR+/HER2− metastatic breast cancer in clinical trials such as the FIRST and the FALCON [13–15]. Chemotherapy is considered an alternative to endocrine therapy and targeted therapy. While combination chemotherapy may offer improved efficacy, it is often associated with higher levels of toxicity. Therefore, sequential monotherapy is a preferred treatment approach. In clinical practice, weighing the toxicity profile against patient benefits is crucial when deciding on chemotherapy. Taxanes, including paclitaxel, nanoparticle albumin-bound paclitaxel, and docetaxel, are commonly used for treating metastatic breast cancer. Capecitabine has demonstrated benefits in multiple Phase 2 metastatic breast cancer clinical trials, with an overall response rate of 28%–30% [16]. Therefore, capecitabine was used for metronomic chemotherapy in this case.

Following a local recurrence in the left breast with multiple bone metastases, the patient achieved a PFS of 39 months. The pathology report of the metastatic lesions showed ER-negative and PR-positive 5%, considering that decalcification of bone lesions may affect protein expression. Additionally, due to the patient's poor tolerance to chemotherapy, the treatment continued to focus on systemic therapy centered on endocrine therapy. While guidelines recommend the use of CDK4/6 inhibitors in combination with endocrine therapy, these drugs were unavailable at our hospital. We used OFS (including medications and oophorectomy) in combination with AIs and then fulvestrant; however, the overall efficacy was unsatisfactory.

In September 2021, the patient developed liver metastasis following progression of bone metastases, with biopsy confirming a triple-negative phenotype. Given the lack of hormone receptor expression and borderline HER2 status, endocrine therapy and HER2-targeted therapy were no longer applicable, prompting a transition to chemotherapy-based treatment. Currently, there is no standardized treatment available for triple-negative breast cancer (TNBC). Responses to treatment in patients with TNBC are often short, followed by rapid relapse, with frequent visceral and brain metastases [17]. Treatment options for patients with advanced TNBC include the antimetabolites capecitabine and gemcitabine, the nontaxane microtubule inhibitor eribulin, and platinum. The median PFS after chemotherapy ranges from 1.7 to 3.7 months [18], while the median overall survival from metastasis ranges from 10 to 13 months [19]. In clinical trials, the median PFS for patients with advanced TNBC receiving taxane or platinum chemotherapy is reported to be 4–6 months, with a median overall survival of 11–17 months [20, 21].

Albumin-bound paclitaxel was selected for systemic therapy based on its established efficacy and tolerability in metastatic TNBC. The patient achieved a PFS of 23 months on this regimen, reflecting a relatively durable response. However, following a brief treatment interruption, tumor markers increased, suggesting disease progression. Given the patient's prior exposure to taxanes and her clinical condition, oral capecitabine was introduced as metronomic chemotherapy, aiming to maintain disease control with a favorable toxicity profile. Local treatment of the metastatic lesions involved hepatic particle implantation followed by metastatic liver cancer palliative high-intensity focused ultrasound therapy, resulting in a reduction of liver lesions compared to previous scans, and liver metastases were stable.

### 3.2. Molecular Typing Changes During Treatment

A total of four pathological biopsies were conducted during the course of the patient's treatment. With disease progression, the pathological features of the patient's breast cancer underwent a shift from HR+ to a TNBC-like phenotype. The primary tumor was ER (+), PR (+), and HER2 (−), while the liver metastasis was ER (−), PR (−), HER2 (1+), and Ki-67 40%, aligning with the characteristics of TNBC (Table 1).

This phenotypic evolution likely reflects clonal selection under therapeutic pressure, where endocrine-sensitive clones are eliminated and therapy-resistant subclones expand. A recent meta-analysis reported discordance rates of 20%, 33%, and 8% for ER, PR, and HER2 expression, respectively, between primary and metastatic lesions, with a notable trend of ER/PR loss during progression, especially in bone and liver metastases [22]. In addition to clonal evolution, epigenetic mechanisms such as ESR1 gene promoter hypermethylation have been implicated in ER loss, particularly in TNBC cases [23]. Moreover, loss of transcription factors like GATA3, which plays a key role in luminal differentiation, may contribute to ER downregulation and tumor dedifferentiation [24]. Changes in the tumor microenvironment, including cytokine signaling and immune cell infiltration, may also influence receptor expression and tumor phenotype [25].

These findings underscore the dynamic nature of breast cancer biology and highlight the need for repeated biopsies and receptor reassessment throughout disease progression to guide optimal treatment strategies.

### 3.3. Bone Metastasis Management—Bone Pseudoprogression

**Figure 2:**
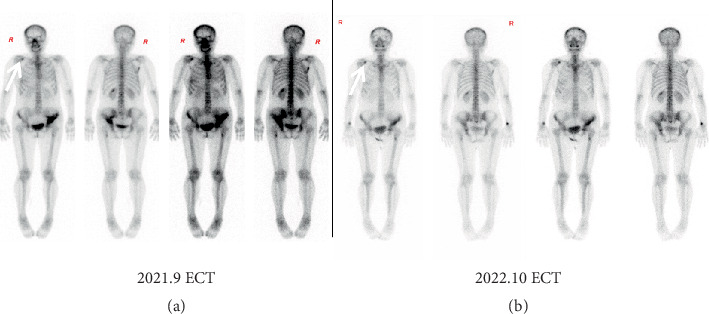
ECT images from September 2021 to October 2022. (a) ECT image from September 2021, taken 1 month after switching to denosumab, shows increased standard uptake value and an increased range of metastases, including new consolidations in the right scapula, multiple areas in the spine, and the femur and left ilium. (b) ECT image from October 2022 indicates no additional bone metastases, and the existing lesions remain unchanged.

Patients with breast cancer and bone metastasis often receive treatment with bone-targeted agents, and radiotherapy may also be considered. The patient initially received zoledronic acid for 2 years. As the bone metastasis progressed and the patient reported bone pain again, the treatment regimen was switched to denosumab, which has been shown to be superior to zoledronic acid in delaying or preventing skeletal-related events in patients with breast cancer bone metastasis and is generally well tolerated [26]. Two months after the switch to denosumab, ECT showed increased metabolic activity in the bone metastases, suggesting possible progression. However, the patient reported reduced bone pain, suggesting that the ECT results may indicate a “bone flare” rather than disease progression. Similar findings have been noted in a previous study as pseudoprogression [27]. Bone pseudoprogression refers to a transient increase in osteoblastic activity that occurs in response to effective treatment. It is frequently observed on bone scans as increased uptake, which can be misinterpreted as disease progression. Clinical symptoms such as pain relief and lack of new lesions on imaging are important clues to differentiate pseudoprogression from true progression. This is further confirmed by the ECT review 1 year later, with no progression of bone lesions and lower uptake (Figure 2a,b). The patient also reported no significant bone pain. Therefore, it is important to note that the increased bone scan uptake value only represents active metabolism, rather than being indicative of disease progression. Pseudoprogression of bone lesions is considered a favorable treatment response that is often seen in patients with breast cancer. In such cases, it is recommended to continue with bone-modifying agents and schedule additional evaluations based on the patient's symptoms.

## 4. Conclusions

In this case report, we detailed the treatment course of a patient with HR+ breast cancer who subsequently developed local recurrence, bone metastases, and visceral metastases after the initial treatment. Notably, the absence of local radiotherapy during the initial treatment may have contributed to the local recurrence. Systemic treatment might yield a more favorable prognosis. Regular follow-up is essential for timely adjustments to treatment regimens tailored to the evolving disease status, particularly in response to pathological changes. A key takeaway from this case is the dynamic nature of tumor biology, as evidenced by the progressive shift in molecular typing toward a TNBC phenotype. This underscores the need for clinicians to remain vigilant about potential molecular evolution and to consider rebiopsy and re-evaluation of molecular markers during disease progression.

Additionally, the case highlights the diagnostic challenge posed by bone pseudoprogression (bone flare), which can mimic disease progression on imaging. In this context, clinical symptoms and follow-up imaging are crucial for differentiating true progression from treatment response. Recognizing pseudoprogression can prevent premature alterations in effective therapies and improve patient outcomes.

Genetic testing may better guide treatment decisions and prognostic predictions. Moreover, the detection of distant metastasis is frequently overlooked during current follow-up procedures for HR+ breast cancer. Given that bone is the most common site of metastasis, regular bone scans are necessary for patients with breast cancer, with denosumab showing superior efficacy in reducing the risk of skeletal-related events. Attentiveness to identifying and managing bone flare is also essential in treating bone metastasis.

## Figures and Tables

**Table 1:** Pathological results.

	**Primary breast cancer**	**Recurrent in the chest wall**	**Bone metastasis**	**Liver metastases**
ER	(++) 50%	(+) 10%	(−)	(−)
PR	(+++) 75%	(−)	(+) 5%	(−)
HER2	(−)	(−)	(−)	(1+)
Ki-67	20%	15%	< 1%	40%

Abbreviations: ER, estrogen receptor; HER2, human epidermal growth factor Receptor 2; PR, progesterone receptor.

## Data Availability

The data are available from the corresponding author on reasonable request.
